# Supplementary material for: Biomineral armor in leaf-cutter ants
Source: Nat Commun. 2020 Nov 24;11:5792. doi: 10.1038/s41467-020-19566-3 (PMC7686325; doi:10.1038/s41467-020-19566-3)
Supplement: Supplementary file 3 — Description of Additional Supplementary Files [file 41467_2020_19566_MOESM3_ESM.docx]

**Description of Additional Supplementary Information**

**Legends for Supplementary Movies 1 to 3:**

**Supplementary Movie 1.** Representative real-time microscopic video of in-situ nanoindentation measurement on Ac. echinatior cuticle.

**Supplementary Movie 2.** The time lapse of aggressive experiments between At. cephalotes soldier and three Ac. echinatior worker ants with mineral-present or absent.

**Supplementary Movie 3.** Details of aggression experiments between At. cephalotes soldier and mineral-free Ac. echinatior worker ants. Note that At. cephalotes soldier cut throughout thorax of mineral-free Ac. echinatior worker ants. We hope that this revised manuscript will be suitable for publication in Nature Communications and look forward to hearing from you in due course. Sincerely, on behalf of all co-authors,
