# Supplementary material for: Biomineral armor in leaf-cutter ants
Source: Nat Commun. 2020 Nov 24;11:5792. doi: 10.1038/s41467-020-19566-3 (PMC7686325; doi:10.1038/s41467-020-19566-3)
Supplement: Supplementary file 7 — Reporting Summary [file 41467_2020_19566_MOESM7_ESM.pdf]

nature research

Corresponding author(s): Cameron R Currie
Last updated by author(s): Sept 8, 2020

Reporting Summary

Nature Research wishes to improve the reproducibility of the work that we publish. This form provides structure for consistency and transparency in reporting. For further information on Nature Research policies, see Authors & Referees and the Editorial Policy Checklist.

Statistics

For all statistical analyses, confirm that the following items are present in the figure legend, table legend, main text, or Methods section.

n/a	Confirmed	
☐	☒	The exact sample size (*n*) for each experimental group/condition, given as a discrete number and unit of measurement
☐	☒	A statement on whether measurements were taken from distinct samples or whether the same sample was measured repeatedly
☐	☒	The statistical test(s) used AND whether they are one- or two-sided<br>*Only common tests should be described solely by name; describe more complex techniques in the Methods section.*
☒	☐	A description of all covariates tested
☒	☐	A description of any assumptions or corrections, such as tests of normality and adjustment for multiple comparisons
☒	☐	A full description of the statistical parameters including central tendency (e.g. means) or other basic estimates (e.g. regression coefficient) AND variation (e.g. standard deviation) or associated estimates of uncertainty (e.g. confidence intervals)
☐	☒	For null hypothesis testing, the test statistic (e.g. *F*, *t*, *r*) with confidence intervals, effect sizes, degrees of freedom and *P* value noted<br>*Give P values as exact values whenever suitable.*
☒	☐	For Bayesian analysis, information on the choice of priors and Markov chain Monte Carlo settings
☒	☐	For hierarchical and complex designs, identification of the appropriate level for tests and full reporting of outcomes
☒	☐	Estimates of effect sizes (e.g. Cohen's *d*, Pearson's *r*), indicating how they were calculated

Our web collection on statistics for biologists contains articles on many of the points above.

Software and code

Policy information about availability of computer code

Data collection	The Igor Pro macros, called GG Macros , used to produce PIC maps are available free of charge on https://home.physics.wisc.edu/gilbert/software/.
Data analysis	The code to measure the angular distances of c-axes in Fig. 2 is available on https://home.physics.wisc.edu/gilbert/software/.

For manuscripts utilizing custom algorithms or software that are central to the research but not yet described in published literature, software must be made available to editors/reviewers. We strongly encourage code deposition in a community repository (e.g. GitHub). See the Nature Research guidelines for submitting code & software for further information.

Data

Policy information about availability of data

All manuscripts must include a data availability statement. This statement should provide the following information, where applicable:
- Accession codes, unique identifiers, or web links for publicly available datasets
- A list of figures that have associated raw data
- A description of any restrictions on data availability

All data is available in the main text or the supplementary materials, and all list of figures that have associated raw data.

Field-specific reporting

Please select the one below that is the best fit for your research. If you are not sure, read the appropriate sections before making your selection.

☐ Life sciences ☐ Behavioural & social sciences ☒ Ecological, evolutionary & environmental sciences

For a reference copy of the document with all sections, see nature.com/documents/nr-reporting-summary-flat.pdf

Ecological, evolutionary & environmental sciences study design

All studies must disclose on these points even when the disclosure is negative.

Study description	We ran ant battle and fungal infection experiments testing the functionality of biominerals, and at least 3 replicates for each experiment were given. All data reported are quantitative. Our ant sampled from Brazil and Costa Rico.
Research sample	Fungus-farming ants including Acromyrmex echinatior and Atta cephalotes from Brazil and Costa Rico, and all the ant colonies were maintained in the lab. Our samples are characterized by overall Acromyrmex echinatior and Atta cephalotes populations.
Sampling strategy	For the battle and infection experiments, we randomly sampled ants from the ant colony, as social insect, fungus-farming ants are genetically identical. For each experiment, at least 3 replicates were provided. Moreover, our ant collections covered an extensive geographic distribution. Together, our sampling give a robust picture of the genomic and/or physiology variability among individuals .
Data collection	All the authors were involved int the data collection and record, in particular Hongjie Li, Chang-yu Sun and Caitlin M. Carlson. Ant nests are collected by excavating the ground and maintain in plastic boxes together with wet cotton.
Timing and spatial scale	From January 1 2019 to May 1 2019 for data collection in UW-Madision.
Data exclusions	No data were excluded from the analyses.
Reproducibility	All the measurements at least in 3 replicates and perform indepedentally
Randomization	We randomly collected ants from the ant colony, as social insect, fungus-farming ants are genetically identical.
Blinding	Not relevant. Our study is to report the discovery the presence of biomineral on the ant cuticle other than comparison.

Did the study involve field work? ☒ Yes ☐ No

Field work, collection and transport

Field conditions	Temperature exceeded 25ºC, wind speed was less than 4 m/ at 1m above ground level, and skies were sunny on all the sampling days.
Location	Ac. echinatior and At. cephalotes collected from Bazail (22°49.886’S 48°25.426’W and 22°54.353’S 48°14.562’W, respectively) and Costa Rica (9°56'07.9"N 84°03'04.4"W).
Access and import/export	Collections of Brazilian samples are approved according to Sistema de Autorização e Informação em Biodiversidade 46555-5 and Conselho Nacional de Desenvolvimento Científico e Tecnológico 010936/2014-9 authorizations. Collection permits were granted by the “Comisión Institucional de Biodiversidad” (Institutional Biodiversity Committee, University of Costa Rica; Resolution 020; Material Transfer Agreement MTA VI-4307-2013) and authorized by La Selva Biological Station.
Disturbance	No disturbance was caused during our sampling.

Reporting for specific materials, systems and methods

We require information from authors about some types of materials, experimental systems and methods used in many studies. Here, indicate whether each material, system or method listed is relevant to your study. If you are not sure if a list item applies to your research, read the appropriate section before selecting a response.

Materials & experimental systems

n/a	Involved in the study	
☒	☐	Antibodies
☒	☐	Eukaryotic cell lines
☒	☐	Palaeontology
☐	☒	Animals and other organisms
☒	☐	Human research participants
☒	☐	Clinical data

Methods

n/a	Involved in the study	
☒	☐	ChIP-seq
☒	☐	Flow cytometry
☒	☐	MRI-based neuroimaging

Animals and other organisms

Policy information about studies involving animals; ARRIVE guidelines recommended for reporting animal research

Laboratory animals	No laboratory animals are involved in this study
Wild animals	Fungus-farming ants including Acromyrmex echinatior and Atta cephalotes
Field-collected samples	Fungus-farming ants including Acromyrmex echinatior and Atta cephalotes maintain in ant room at 25 oC.

Ethics oversight	At our knowledge, no ethical approval or guidance was required to use these ants.

Note that full information on the approval of the study protocol must also be provided in the manuscript.
