# Supplementary material for: Biomineral armor in leaf-cutter ants
Source: Nat Commun. 2020 Nov 24;11:5792. doi: 10.1038/s41467-020-19566-3 (PMC7686325; doi:10.1038/s41467-020-19566-3)
Supplement: Supplementary file 2 — Peer Review File [file 41467_2020_19566_MOESM2_ESM.pdf]

Reviewer #1 (Remarks to the Author):

Biomineral armor in leaf-cutter ants – Review

I. reviewer's opinion

The quality of the manuscript meets the requirements for publication, under revision and additional experiments

II. Reviewer's general remark

From what I can tell, the manuscript is technically sound and clearly written. Subtleties of electron microscopy and crystallography are inherently difficult to grasp for a non-specialist audience, and the authors made a point to try to explain them thoroughly. Rearing experiments were mostly performed according to expected standards. The findings presented are novel and exciting, shedding some light on new aspects of leaf-cutter ants' functional morphology. Overall, this is a good paper that deserves consideration. However, several major problems remain regarding experimental framing, data acquisition, handling, and presentation. I also want to suggest corrective/additional experiments to strengthen the authors' evolutive interpretations that, in the present form, fail to consider key aspects of ants' functional morphology. Finally, it needs to be stated out that the manuscript would benefit from putting results in a broader perspective and reflect further with the relevant insect literature.

III. Major remarks

To the best of my knowledge, I have no reason to question the first part of the major findings of this study, describing a high Mg-calcite biomineral depot overlaying the ant's cuticle, which is significantly hardened accordingly. This discovery alone is worth congratulating authors for their analysis.

I do however express some restrain when it comes to the evolutive claims made in the manuscript. Since authors partially investigated two obvious potential drivers of biomineralization (interspecific aggression and immunity), it strikes me that they did not consider water loss and protection against phorids as third and fourth. In the present form of the manuscript, authors argue that violent interactions are the main driver behind the biomineralization of A. echinatior's exoskeleton, while they omitted to test properly for two other major potential hypotheses. They framed their analysis and interpretations around the idea that competition for resources with Atta colonies and predation by raider ants should be intense, mostly centring their argument later on around violent interactions with Atta soldiers in the manuscript (Line 79-85, 237-243, 281-292). Survival to violent interactions undeniably seems to be increased because of this biomineralization phenomena, however natural history of ants suggest that its potential role is likely not as clear as the authors want to claim in their present conclusions.

(1) Violent interactions

First, Acromyrmex and Atta have divergent nesting habits, and two genera tend to show differences in climatic preferences (Acromyrmex species tend nest in drier areas than Atta does, Pers. Obs). The two genera often show little overlap at the local scale alongside large parts of their distribution (Wetterer, 1995). Whether this is the direct result of differential climatic requirements, or as Fowler suggested a direct result of inter-generic exclusion through intense competition, is currently unresolved and most likely case dependent (Fowler, 1983; Wetterer, 1995). Authors should refer to this additional literature to nuance their introduction on aggressive competitions between their two model species.

Second, most species of ants experience arguably violent to entirely warfare-focused lifestyles (including but not limited to, true raider ants from the subfamilies Ecitoninae and Dorylinae, Ophthalmopone sp., Megaponera analis, and several Carebara sp). If surviving violent encounters with con or interspecific workers was the sole driver of the biomineralization observed in A. echinatior, one could expect it to be more common across ant taxa, including other fungus-growing ants that are, as the manuscript points out, prime targets for raiding. So far, authors show themselves that this is not the case for Atta, which is the sister genus of Acromyrmex.

It may be that such bio mineralisation in indeed a direct consequence of violent altercations' pressure and has yet to be described in other species exposed to similar pressures. It could be that this is a specific response of some species of Acromyrmex to balance specific lifestyles (smaller colonies than Atta sp., leading to higher investment into protecting the foraging individual itself). In any case, authors should use caution and include sections in their discussion reflecting the only partial understanding of the potential evolutive drivers behind biomineralization that their data can provide.

(2) Water loss

Acromyrmex species tend to nest in drier areas than Atta does, and support lower humidity in laboratory conditions as well. Some lower Attine seem also particularly drought resistant, for terrestrial ants (Hood & Tschinkel, 1990). It cannot be ruled out at this stage that a third potential major driver of biomineralization would be reduced water loss, which could support the observed Acromyrmex ecology. Most desiccation resistant ant species achieve so by modulating their layer of cuticular hydrocarbons (Gibbs, 1998; Hood & Tschinkel, 1990). Biomineralization on the other hand, could represent a truly unique adaptation stemming from the need to balance several pressures at once (enhanced exoskeleton mechanical properties as well as reduced water loss). Because addressing this hypothesis is both easy and could bear results shifting around our entire understanding of this newly described structure, it needs to be tested to meet with the quality standards of a high-profile journal.

IV. Corrections/additions to current experiments to support author's interpretations

(a) Nanomechanical testing

In its current form, the nanomechanical test (Fig 3.a) lacks an appropriate control group. Without including a third species of ant from the Myrmicinae subfamily in their analysis (to which both Atta

and Acromyrmex belong), current result should be interpreted as a bispecies comparison only, in which case it is impossible to tell if biomineralization truly provide Acromyrmex with an evolutive advantage as compared to its relatives. In other words, Atta could be the outlayer among Myrmicinae (with a comparatively weak exoskeleton), which is something authors should correct their experimental design for with adequate ant controls. Data provided for other insect families, albeit very informative, still fails in this regard. Since Myrmicinae have, among ants, rather thick exoskeletons (Peeters et al., 2017), it is crucial for the third control species to be a non-biomineralized, close relative of Acromyrmex from this subfamily. Since Author's claim that ''many fungus growing ants are variably coated with a whitish granular coating'' (Line 86-87), this matter would be elegantly resolved by including data for both a lower Attine, such as Trachymyrmex sp., and a Myrmicinae outgroup species, such as Solenopsis invicta.

(b) Entomopathological fungi infections

In its current form, the metarhizium infection's survival experiment lacks an appropriate control group. Since experiment design only tested for biomineralized vs non-biomineralized Acromyrmex workers, it fails to address the fundamental evolutive point it was aimed to (i.e. to test whether or not biomineralization would provide a competitive advantage as compared to other species of ants lacking such a structure). I would suggest including Atta workers' survival rates as controls in the experiment.

(c) Cuticular water loss assays

Based on Acromyrmex ecology, enhanced resistance to cuticular water loss is a hypothesis that needs to be considered equally with violent aggressions and immunity. Author's need to assess the rate and severity of water loss in biomineralized versus non biomineralized A. echinatior workers, using Atta as control. This could be easily achieved using flow-through respirometry to measure real time water vapor emission (See Schilman et al., 2005 for methods), or for efficiency sake, more crudely by assessing survival rates and LT50 of workers exposed to a low humidity environment (See Hood & Tschinkel, 1990).

.

(d) Protection against parasitoids

It is worth mentioning that protection against phorids could also be a likely driver underpinning biomineralization. While including this hypothesis in their experimental design would have been ideal for author to interpret their data in an evolutive context, I understand that doing so would prove difficult and time consuming at this point. Nevertheless, the suggestion remains.

As reviewer, I'd like to point out that these would either bring strong support the current authors' claims that biomineralization is mainly driven by aggressive interactions or allow to nuance results with a careful approach for immunity/water loss. In any cases, this would greatly improve the evolutive aspect of the results presented in the manuscript and provide the possibility for both authors and readers to make more adequate interpretations of their findings.

V. Minor remarks

Introduction

Line 71: A mature leaf-cutter ant colony comprises a "superorganism" with 100,000 to > 5 million workers, a single queen.

Acromyrmex colonies have been showed to both be smaller (Wetterer, 1995) and, for some species, including A. echinatior which is the focus of the present study, facultatively polygyneous (Bekkevold et al., 1995). Please nuance.

Line 82 – 84: Smaller fungus-growing ant colonies are also subject to attack by the large-sized soldier castes of Atta leaf-cutter ants, which use their powerful mandibles to defend their colonies' territories against other, encroaching ant species

Please refer to major remark (1) to nuance that violent competition with Atta is not always a rule in smaller fungus growing species

Line 86: Many species of fungus-growing ants are variably covered with a whitish granular coating, uniformly distributed on their otherwise dark brown cuticles

Please give a few examples.

General: please include and cite the ant functional morphology relevant literature to broaden the introduction’s scope:

- In the case of leaf-cutting ant, Zinc and Manganese incorporation was showed to enhance hardness of the mandibular cuticle in mature worker (Schofield et al., 2002).

- Metal is incorporated to harden the sting cuticle of aculeate Hymenoptera, including ants (Baumann et al., 2018)

- A whitish/silver coating was also found in the Sahara silver ant Cataglyphis bombycina, with relation to reflecting sunlight through total internal reflection (Shi et al., 2015; Willot et al., 2016).

Results and discussion

Line 212-215: Please frame results of cuticle thickness in their broader evolutionary perspective in ants using data from Peeters et al., 2017. It might be worth mentioning that some subfamilies (Ponerine) do have even thicker exoskeletons than Acromyrmex, and likely harder too as a result, but no evidence points out toward biomineralization in those species so far.

Line 230, Fig 3C, supp. Fig 19: Please quickly explain in methods the procedures used to count body parts. Please provide adequate statistical analysis of data

Line 231, Fig. 3d: Low sample size for Atta worker survival (N=5, Line 542-546) and high sample size asymmetry (N=15 for Acromyrmex). Experiment is easily replicable, please reach comparable sample size between Acromyrmex and Atta to ensure sufficient statistical power.

Line 255, Fig. 3e: Please provide adequate statistical analysis of data .

Line 280 – 292: This is a strong statement. Such parallel with human cultural evolution would be appropriate if a compelling case was made in favour of a protective and defensive role of biomineralization in A. echinatior. Unfortunately, this is not yet entirely the case, and authors should thus be weary not to jump to conclusions their data do not fully support. Please keep in the form if additional experiments provide ample support for protection. Adapt and use caution otherwise in the final form of the manuscript.

VI. References

• Baumann, K., Vicenzi, E. P., Lam, T., Douglas, J., Arbuckle, K., Cribb, B., ... & Fry, B. G. (2018). Harden up: metal acquisition in the weapon

• Bekkevold, D., Frydenberg, J., & Boomsma, J. J. (1999). Multiple mating and facultative polygyny in the Panamanian leafcutter ant Acromyrmex echinatior. Behavioral ecology and sociobiology, 46(2), 103-109.

• Gibbs, A. G. (1998). Water-proofing properties of cuticular lipids. American Zoologist, 38(3), 471-482.

• HOOD, W. G., & TSCHINKEL, W. R. (1990). Desiccation resistance in arboreal and terrestrial ants. Physiological Entomology, 15(1), 23-35

• Peeters, C., Molet, M., Lin, C. C., & Billen, J. (2017). Evolution of cheaper workers in ants: a comparative study of exoskeleton thickness. Biological Journal of the Linnean Society, 121(3), 556-563.

• Schilman, P. E., Lighton, J. R., & Holway, D. A. (2005). Respiratory and cuticular water loss in insects with continuous gas exchange: comparison across five ant species. Journal of Insect Physiology, 51(12), 1295-1305.

• Schofield, R. M., Nesson, M. H., & Richardson, K. A. (2002). Tooth hardness increases with zinc-content in mandibles of young adult leaf-cutter ants. Naturwissenschaften, 89(12), 579-583.

• Shi, N. N., Tsai, C. C., Camino, F., Bernard, G. D., Yu, N., & Wehner, R. (2015). Keeping cool: Enhanced optical reflection and radiative heat dissipation in Saharan silver ants. Science, 349(6245), 298-301.

• Wetterer, J. K. (1995). Forager size and ecology of Acromyrmex coronatus and other leaf-cutting ants in Costa Rica. Oecologia, 104(4), 409-415.

• Willot, Q., Simonis, P., Vigneron, J. P., & Aron, S. (2016). Total internal reflection accounts for the bright color of the Saharan silver ant. PloS one, 11(4).

Reviewer #2 (Remarks to the Author):

This manuscript discusses the compelling, novel finding that Acromyrmex echinatior leaf cutting ants biosynthesize high-magnesium calcite. This remarkable discovery is surely to be of interest andelevance to the broad scientific readership of Nat Comm. Though I am not familiar with the majority of techniques used in the work presented, I found this manuscript easy to read and convincing since the authors addressed their findings from multiple, complimentary angles, with seemingly suitable positive and negative controls, that each reinforced their conclusions independently.

I do, however, have some comments, questions and suggestions about the experiments that assess the potential adaptive functions of the magnesium calcite armor discovered.

Both the aggression experiments and the infection experiments test if there is a difference in survival between ants with and without biomineral armor within the same Atta species. I am assuming that these tests make use of younger ants (0-6 days old) and older ants (6-8 days old), but this isn't specified anywhere. I would, therefore, like to suggest that at least more details are provided about the ants in the with and without biomineral armor groups.

Assuming that the with and without biomineral armor groups consist of younger vs older ants, I am wondering if the differences in survival as a result of the aggression experiments is not entirely attributable to their armor but might at least be partially due to behavioral differences in aggression between the two age groups. I think the authors should at least discuss this possibility. Especially if there is behavioral evidence (either from their own experiments or from the literature) that shows that aggression levels are similar between younger and older ants since this would make the case they are trying to make stronger.

Similarly, I am wondering if age effects are playing a role as well in the infection experiments? Could there be a difference in grooming behavior, immune response or density of antibiotic-producing bacteria on the exoskeleton that could partially play a role in the difference of survival observed between the with and without biomineral groups? Again, I am of the opinion that the authors should include some sort of discussion about the potential roles that factors outside of calcification could play here.

Not to say that the authors should do additional experiments per se, but would it be possible to feed their ants bacteria-specific antibiotics to rid them of symbiotic bacteria on the exoskeleton? Could they include an ant species in their infection experiment, such as At. Cephalotus that doesn't have a biomineralized exoskeleton to see if their survival curve is more similar to "without" At. Echinatior group to provide more evidence for the exoskeleton causing the difference here? Could they compare infected ants kept in solitude versus those kept in groups of three to rule out differences in grooming effects?

And, perhaps not the toughness caused by biomineralization but potential differences in hydrophobicity of the exoskeletons cause spores to adhere less well in the "with" group compared to the "without" group. Could hydrophobicity be tested?

Just some ideas if the authors would be interested in exploring further down this road to get more specific answers.

From the fact that the infection experiments are not included in the final two concluding paragraphs of the main text, I am assuming that the authors indeed did not find their infection experiments easy to conclusively interpret either. However, I would like to suggest to perhaps add some sort of discussion/conclusion on this aspect here.

As for my comment based on grooming behavior, this comes forth from the confusion I had, while reading the materials and methods for the infections experiment. I would suggest revising Lines 562-563 to clarify better how many ants were in each group, how many replicates there were for each group (I think 5? From 5 subcolonies?) and if these ants were kept in groups of three (meaning they could groom one another to remove spores) or by themselves (meaning it would be more difficult to groom).

Further reading the methods, it also became clear that there were control groups with sham treatments. These groups should be described better (I assume a control for both with and without biomineralization was performed for every replicate but this is not clear) and should be included in the survival curve in Figure 3.

As for that survival curve. It suggests that not all ants with biomineralization died within 6 days post infection. However, the main text (Line 255) mentions that all ants died within 6 days. Maybe I am misinterpreting Lines 251-255. Could the authors revise these lines to make them more clear?

Additionally, the authors should provide more information about the fungal aspects of their infection experiment. The strain number of Metarhizium anisopliae should be mentioned. Also, the authors might want to check if this strain is still referred to as M. anisopliae since many isolates have been recognized to be specific and have, as such, have been assigned their own species names.

The authors should also include culturing conditions including media used, time cultured, at what temperature, and if spores were harvested fresh before each infection experiment to assure that spores were equally viable for each replication and each group tested (did they do a spore viability test perhaps?).

Moreover, the authors mention that fungal growth was observed from cadavers of succumbed ants without biomineralization but not with. How long were these ant cadavers incubated? Under which conditions? And how many cadavers of those incubated showed growth for each group? Did they find 100% growth in cadavers without and 0% growth in cadavers with biomineralization, even though they were incubated for the same amount of time under the same conditions? This information will provide the necessary context to interpret the results that the authors report on better.

Despite these points, that I feel the authors should at least consider addressing, I really enjoyed reading this manuscript because of the incredibly exciting findings reported.

Reviewer #3 (Remarks to the Author):

Li et al's article describes an investigation into the structure of the epicuticle of leaf-cutter ants. The authors show that it is covered with a thin layer of high Mg calcite, and that the formation of this phase is promoted by the organic matrix. The mineralized layer is shown to offer superior mechanical properties. I thoroughly enjoyed reading this article, where it was such a nice change to read a simple story that was so easy to follow. And I loved how the story built to an exciting climax where the importance of superior mechanical properties are tested when the ants go to war and encounter a fungus plague. I think this is an important first demonstration of biomineralization in the insect world, and I recommend publication subject to some minor changes.

1. Sometimes the authors use terminology that I was not familiar with/ is specific to the field of "ants". As this article will also be read more generally, the authors need to explain any specific terms. Some I spotted were "obligate mutualism", "eclosion" and "callow".

2. In the section "Morphological, structural, and chemical characteristics of epicuticular minerals" please provide a better description of the crystals (size, shape, thickness of the crystal layer etc). Currently, one has to look at the Figs to find this. It would also be useful to give information on what % of the cuticle corresponds to the mineral layer.

3. In Fig 1 there is a huge image of an ant (I roughly know what these look like) but the image of the mineral film - which is the focus of the paper - is really small.

4. Fig 1b. Is the reference calcite diffractogram pure calcite? There doesn't seem to be a good fit to either calcite or dolomite. It would be more helpful if a Mg-calcite reference was used, giving a perfect match to the experimental peaks.

5. Fig 1h is really small such that I find it hard to see the detail in the images.

6. I found Figs S1 and S2 really useful in helping me to visualize the system. Please move to the main paper (you have space).

7. Characterization of the mineral layer is quite difficult as it is so thin compared with the cuticle. Your data is convincing that you have a high Mg calcite. However, is it not possible that an amorphous phase is also present? This would not be surprising at such high Mg levels, and the composition of the mineral layer is likely to depend on its maturity. If so, the crystalline peaks would be weaker from a given volume of sample than if it is purely crystalline. Looking at your data it is possible that this is the case. Morphologically, the sample does not look purely crystalline, and the TEM also looks like it is a mixture.

8. The authors often refer to “no observable Ca-Mg ordering”. As far as I am aware the only Ca/Mg system where there is ordering is dolomite which is a specific phase. So, do they mean that they see no evidence of dolomite?

9. Considering the ability of the epicuticle to promote the formation of high Mg calcite rather than calcite, is this due to nucleation on the specific surface, or soluble proteins released into the solution?

Author’s Response to Reviewer 1:

Biomineral armor in leaf-cutter ants – Review

I. reviewer’s opinion
The quality of the manuscript meets the requirements for publication, under revision and additional experiments

We thank the reviewer for the encouraging comments on our work.

. Reviewer’s general remark
From what I can tell, the manuscript is technically sound and clearly written. Subtleties of electron microscopy and crystallography are inherently difficult to grasp for a nonspecialist audience, and the authors made a point to try to explain them thoroughly. Rearing experiments were mostly performed according to expected standards. The findings presented are novel and exciting, shedding some light on new aspects of leaf-cutter ants’ functional morphology. Overall, this is a good paper that deserves consideration.

We thank the reviewer for the positive words relating to our work and manuscript.

However, several major problems remain regarding experimental framing, data acquisition, handling, and presentation. I also want to suggest corrective/additional experiments to strengthen the authors’ evolutive interpretations that, in the present form, fail to consider key aspects of ants’ functional morphology. Finally, it needs to be stated out that the manuscript would benefit from putting results in a broader perspective and reflect further with the relevant insect literature.

We will address these points under the ‘major remarks’ section below.

. Major remarks
To the best of my knowledge, I have no reason to question the first part of the major findings of this study, describing a high Mg-calcite biomineral depot overlaying the ant’s cuticle, which is significantly hardened accordingly. This discovery alone is worth congratulating authors for their analysis.

Again, thanks for the encouraging comments on our work!

I do however express some restrain when it comes to the evolutive claims made in the manuscript. Since authors partially investigated two obvious potential drivers of biomineralization (interspecific aggression and immunity), it strikes me that they did not consider water loss and protection against phorids as third and fourth. In the present form of the manuscript, authors argue that violent interactions are the main driver behind the biomineralization of A. echinatior’s exoskeleton, while they omitted to test properly for two other major potential hypotheses. They framed their analysis and interpretations around

the idea that competition for resources with Atta colonies and predation by raider ants should be intense, mostly centring their argument later on around violent interactions with Atta soldiers in the manuscript (Line 79-85, 237-243, 281-292). Survival to violent interactions undeniably seems to be increased because of this biomineralization phenomena, however natural history of ants suggest that its potential role is likely not as clear as the authors want to claim in their present conclusions.

(1) Violent interactions
First, Acromyrmex and Atta have divergent nesting habits, and two genera tend to show differences in climatic preferences (Acromyrmex species tend nest in drier areas than Atta does, Pers. Obs). The two genera often show little overlap at the local scale alongside large parts of their distribution (Wetterer, 1995). Whether this is the direct result of differential climatic requirements, or as Fowler suggested a direct result of inter-generic exclusion through intense competition, is currently unresolved and most likely case dependent (Fowler, 1983; Wetterer, 1995). Authors should refer to this additional literature to nuance their introduction on aggressive competitions between their two model species.

This is a good point. Yes, it is true that *Atta* and *Acromyrmex* can have divergent nesting habits in some regions where they are distributed but, as noted by the reviewer, this is ‘case dependent’. In our case, *Atta cephalotes* and *Acromyrmex echinatior,* used in our study, co-occur in the same nesting areas in Panama (Schultz and Currie personal observations, based on several decades of field work). Further, there is an extensive literature indicating that they co-occur in other regions as well (Fernández and Sendoya 2004 *Biota Colombiana* 5(1) 3-93; Kempf 1972 *Studia Entomologica* 15: 3-344; Brandão Carlos 1991 *Revista Brasileira de Biologia* 35 (2): 319-412.) and personal observations (both coauthors Schultz and Currie have decades of field work experience with leaf-cutter ants). We have added information on this to the methods and appreciate this suggestion from the reviewer (lines 305-308, P.15).

Second, most species of ants experience arguably violent to entirely warfare-focused lifestyles (including but not limited to, true raider ants from the subfamilies Ecitoninae and Dorylinae, Ophthalmopone sp., Megaponera analis, and several Carebara sp). If surviving violent encounters with con or interspecific workers was the sole driver of the biomineralization observed in A. echinatior, one could expect it to be more common across ant taxa, including other fungus-growing ants that are, as the manuscript points out, prime targets for raiding. So far, authors show themselves that this is not the case for Atta, which is the sister genus of Acromyrmex.

We do not believe we state that a ‘violent’ warfare-focused lifestyle is the sole driver of biomineralization. In fact, we present evidence that the benefit incurred from the biomineral armor could include protection from fungal pathogens. We have clarified this in several places, including on lines 102-104, P. 6 of the introduction. Please note that the use of the word ‘armor’ does not necessarily imply ‘warfare’ benefits. The Merriam-Webster Dictionary defines 'armor' as ‘a protective outer layer’. We carefully examined our manuscript to ensure that this is clear.

It may be that such bio mineralisation in indeed a direct consequence of violent altercations' pressure and has yet to be described in other species exposed to similar pressures.

We agree that biomineral armor may be more widespread in ants and other insects than is currently recognized, and specifically address this in our paper (lines 282). But, as we also point out, the formation of high-magnesium calcite is rare in the biosphere, suggesting that it is unlikely to be common in ants and other insects.

It could be that this is a specific response of some species of Acromyrmex to balance specific lifestyles (smaller colonies than Atta sp., leading to higher investment into protecting the foraging individual itself). In any case, authors should use caution and include sections in their discussion reflecting the only partial understanding of the potential evolutive drivers behind biomineralization that their data can provide.

As suggested, we added "and explore two of several possible benefits associated with the biomineral armor in experimental ant battles and infections by entomopathogenic fungi." (lines 98-100, P. 4), and "at least in part, both to (i)......and (ii) protect them from disease organisms that might otherwise spread rapidly in their densely populated colonies." (lines 302-303, P.14).

(2) Water loss
Acromyrmex species tend to nest in drier areas than Atta does, and support lower humidity in laboratory conditions as well. Some lower Attine seem also particularly drought resistant, for terrestrial ants (Hood & Tschinkel, 1990). It cannot be ruled out at this stage that a third potential major driver of biomineralization would be reduced water loss, which could support the observed Acromyrmex ecology. Most desiccation resistant ant species achieve so by modulating their layer of cuticular hydrocarbons (Gibbs, 1998; Hood & Tschinkel, 1990). Biomineralization on the other hand, could represent a truly unique adaptation stemming from the need to balance several pressures at once (enhanced exoskeleton mechanical properties as well as reduced water loss). Because addressing this hypothesis is both easy and could bear results shifting around our entire understanding of this newly described structure, it needs to be tested to meet with the quality standards of a high-profile journal.

We thank the reviewer for pointing out that there are other possible functions of the biomineral armor, including the reduction of water loss, and we agree that there may be other functions. Our experiments simply show two possible functions – not all possible functions. Due to restrictions associated with COVID-19 we are not able to conduct the experiments suggested by this reviewer, so we must leave this to future work. We added the following wording to our introduction: "and explore two of several possible benefits associated with the biomineral armor in experimental ant battles and infections by entomopathogenic fungi." (lines 102-104, P.4). We did not specifically add water loss or other possible functions to the paper because we hesitate to speculate without data.

IV. Corrections/additions to current experiments to support author's interpretations (a) Nanomechanical testing

In its current form, the nanomechanical test (Fig 3.a) lacks an appropriate control group. Without including a third species of ant from the Myrmicinae subfamily in their analysis (to which both Atta and Acromyrmex belong), current result should be interpreted as a bispecies comparison only, in which case it is impossible to tell if biomineralization truly provide Acromyrmex with an evolutive advantage as compared to its relatives. In other words, Atta could be the outlayer among Myrmicinae (with a comparatively weak exoskeleton), which is something authors should correct their experimental design for with adequate ant controls. Data provided for other insect families, albeit very informative, still fails in this regard. Since Myrmicinae have, among ants, rather thick exoskeletons (Peeters et al., 2017), it is crucial for the third control species to be a non-biomineralized, close relative of Acromyrmex from this subfamily. Since Author's claim that ''many fungus growing ants are variably coated with a whitish granular coating" (Line 86-87), this matter would be elegantly resolved by including data for both a lower Attine, such as Trachymyrmex sp., and a Myrmicinae outgroup species, such as Solenopsis invicta.

We are grateful for the reviewer's interest in ant cuticular hardness and we acknowledge that there is a great amount of interesting additional data for us to collect and explore but, again, gathering and adding more information to the present paper is not possible due to COVID19. We also respectfully point out that the reviewer must have missed that we present *Ac. echinatior* workers without the biomineral layer as our main control group (Fig. 4a). We have made this clearer by pointing it specifically out in the introduction: 'We measure the cuticle hardness of A. echinatior ants with and without the cuticular layer using *in-situ* nanoindentation...' (lines 101102, P.5). The use of additional insects as well as *Atta* provide further support that *Ac. echinatior* workers lacking biomineral armor have a similar degree of hardness to other insects (the other insect cuticles studied are similar in hardness, typically around 0.7 Gpa). When the COVID-19 pandemic is over, we look forward to further studies of more ants.

(b) Entomopathological fungi infections

In its current form, the metarhizium infection's survival experiment lacks an appropriate control group. Since experiment design only tested for biomineralized vs non-biomineralized Acromyrmex workers, it fails to address the fundamental evolutive point it was aimed to (i.e. to test whether or not biomineralization would provide a competitive advantage as compared to other species of ants lacking such a structure). I would suggest including Atta workers' survival rates as controls in the experiment.

We agree with the reviewer that it would be desirable to include *Atta* workers in entomopathogenic fungal infection experiments, but due to COVID-19 we are not able to conduct more experiments at this time, so future work will follow up on this. We also point out that our goal was not to determine whether the biomineral armor provides a competitive advantage to a species with biomineral armor vs. a second species without armor, but rather whether it confers protection from entomopathogenic fungi in *Acromyrmex echiniator*. Further, for evolution by natural selection to favor the biomineral armor, the competitive dynamic would occur between conspecifics with less biomineral or no biomineral, not between *Acromyrmex* and *Atta,* because selection acts on conspecific individuals, not on ecological communities of organisms.

(c) Cuticular water loss assays

Based on Acromyrmex ecology, enhanced resistance to cuticular water loss is a hypothesis that needs to be considered equally with violent aggressions and immunity. Author's need to assess the rate and severity of water loss in biomineralized versus non biomineralized A. echinatior workers, using Atta as control. This could be easily achieved using flow-through respirometry to measure real time water vapor emission (See Schilman et al., 2005 for methods), or for efficiency sake, more crudely by assessing survival rates and LT50 of workers exposed to a low humidity environment (See Hood & Tschinkel, 1990).

Again, we are grateful for this suggestion and, as noted above, future work will explore this possibility.

(d) Protection against parasitoids
It is worth mentioning that protection against phorids could also be a likely driver underpinning biomineralization. While including this hypothesis in their experimental design would have been ideal for author to interpret their data in an evolutive context, I understand that doing so would prove difficult and time consuming at this point. Nevertheless, the suggestion remains.

Thanks for this suggestion; future work will explore this.

V. Minor remarks

Introduction
Line 71: A mature leaf-cutter ant colony comprises a "superorganism" with 100,000 to > 5 million workers, a single queen. Acromyrmex colonies have been showed to both be smaller (Wetterer, 1995) and, for some species, including A. echinatior which is the focus of the present study, facultatively polygyneous (Bekkevold et al., 1995). Please nuance.

As suggested, we add "including those of *Acromyrmex*," (lines 86-87, P.5) into introduction.

Line 82 – 84: Smaller fungus-growing ant colonies are also subject to attack by the large-sized soldier castes of Atta leaf-cutter ants, which use their powerful mandibles to defend their colonies' territories against other, encroaching ant species. Please refer to major remark (1) to nuance that violent competition with Atta is not always a rule in smaller fungus growing species.

As suggested, we add "occasionally" (line 87, P.5) into introduction.

Line 86: Many species of fungus-growing ants are variably covered with a whitish granular coating, uniformly distributed on their otherwise dark brown cuticles. Please give a few examples.

As suggested, we add ", including *Acromyrmex echinatior*" (lines 91-92, P.5) into introduction.

General: please include and cite the ant functional morphology relevant literature to broaden the introduction's scope:
- In the case of leaf-cutting ant, Zinc and Manganese incorporation was showed to enhance

hardness of the mandibular cuticle in mature worker (Schofield et al., 2002).
- Metal is incorporated to harden the sting cuticle of aculeate Hymenoptera, including ants (Baumann et al., 2018)
- A whitish/silver coating was also found in the Sahara silver ant Cataglyphis bombycina, with relation to reflecting sunlight through total internal reflection (Shi et al., 2015; Willot et al., 2016).

We thank the reviewer for these references and have cited only those that are relevant to the current study. We add “zinc-enriched....” (line 88, P.5) into introduction.

Results and discussion

Line 212-215: Please frame results of cuticle thickness in their broader evolutionary perspective in ants using data from Peeters et al., 2017. It might be worth mentioning that some subfamilies (Ponerine) do have even thicker exoskeletons than Acromyrmex, and likely harder too as a result, but no evidence points out toward biomineralization in those species so far.

As noted above, we are grateful for the reviewer's interest in ant cuticular hardness and thickness; future work will explore this.

Line 230, Fig 3C, supp. Fig 19: Please quickly explain in methods the procedures used to count body parts. Please provide adequate statistical analysis of data

As suggested, we now added “Counting of lost body parts was based on the video record.” (lines 574-575, P.21).

Line 231, Fig. 3d: Low sample size for Atta worker survival (N=5, Line 542-546) and high sample size asymmetry (N=15 for Acromyrmex). Experiment is easily replicable, please reach comparable sample size between Acromyrmex and Atta to ensure sufficient statistical power.

There is no asymmetry in our experiment. Sorry this was not clear. We present the survivorship based on the percentage, with both sample sizes being N=5, which is the number of replicates conducted. The N=15 the reviewer is referring to is based on 5 experiments involving 3 *Acromyrmex* workers. Given the low variance between replicates within treatment group, and vastly different survival between treatment groups, we believe our results are very robust (as indicated by the statistics, see lines 835-836), and is further corroborated by our supporting video.

Line 255, Fig. 3e: Please provide adequate statistical analysis of data.

As suggested, we now added “(P = 0.05; two-sample t test)” (line 262, p.12).

Line 280 – 292: This is a strong statement. Such parallel with human cultural evolution would be appropriate if a compelling case was made in favour of a protective and defensive role of biomineralization in A. echinatior. Unfortunately, this is not yet entirely the case, and authors should thus be weary not to jump to conclusions their data do not fully

support. Please keep in the form if additional experiments provide ample support for protection. Adapt and use caution otherwise in the final form of the manuscript.

As suggested, we add "at least in part, both to (i)……and (ii) protect them from disease organisms that might otherwise spread rapidly in their densely populated colonies." (lines 302-303, P.14) to tone down this statement.

Author's Response to Reviewer 2:

This manuscript discusses the compelling, novel finding that Acromyrmex echinatior leaf cutting ants biosynthesize high-magnesium calcite. This remarkable discovery is surely to be of interest and elevance to the broad scientific readership of Nat Comm. Though I am not familiar with the majority of techniques used in the work presented, I found this manuscript easy to read and convincing since the authors addressed their findings from multiple, complimentary angles, with seemingly suitable positive and negative controls, that each reinforced their conclusions independently.

We thank the reviewer for the encouraging comments on our work.

I do, however, have some comments, questions and suggestions about the experiments that assess the potential adaptive functions of the magnesium calcite armor discovered.

Both the aggression experiments and the infection experiments test if there is a difference in survival between ants with and without biomineral armor within the same Atta species. I am assuming that these tests make use of younger ants (0-6 days old) and older ants (6-8 days old), but this isn't specified anywhere. I would, therefore, like to suggest that at least more details are provided about the ants in the with and without biomineral armor groups.

We agree with the reviewer that if we had simply used younger versus older ants in this experiment this would be an important concern. However, we did not. The biomineral free workers were in fact reared under conditions where they will not form the biomineral as 'older' ants (see methods lines 512-526, P.20). Thus, this experiment is not confounded by age of worker.

Assuming that the with and without biomineral armor groups consist of younger vs older ants, I am wondering if the differences in survival as a result of the aggression experiments is not entirely attributable to their armor but might at least be partially due to behavioral differences in aggression between the two age groups. I think the authors should at least discuss this possibility. Especially if there is behavioral evidence (either from their own experiments or from the literature) that shows that aggression levels are similar between younger and older ants since this would make the case they are trying to make stronger.

We thank the reviewer for pointing this out. As mentioned above, this experiment did not involve the use of young versus old workers, which would be a concern.

Similarly, I am wondering if age effects are playing a role as well in the infection experiments? Could there be a difference in grooming behavior, immune response or density of antibiotic-producing bacteria on the exoskeleton that could partially play a role in the difference of survival observed between the with and without biomineral groups? Again, I am of the opinion that the authors should include some sort of discussion about the potential roles that factors outside of calcification could play here.

Again, as previously noted, age of worker is not a confounded factor in this experiment. Having said this, the presence of the antibiotic producing bacteria could, in particular, does appear to be correlated with the biomineral and could be playing a role. We have added additional discussion about the potential role of antibiotic-producing *Pseudonocardia* bacterial symbionts in the observed difference in survival, lines 264-267, P.12.

Not to say that the authors should do additional experiments per se, but would it be possible to feed their ants bacteria-specific antibiotics to rid them of symbiotic bacteria on the exoskeleton? Could they include an ant species in their infection experiment, such as At. Cephalotus that doesn't have a biomineralized exoskeleton to see if their survival curve is more similar to "without" At. Echinatior group to provide more evidence for the exoskeleton causing the difference here? Could they compare infected ants kept in solitude versus those kept in groups of three to rule out differences in grooming effects?
And, perhaps not the toughness caused by biomineralization but potential differences in hydrophobicity of the exoskeletons cause spores to adhere less well in the "with" group compared to the "without" group. Could hydrophobicity be tested?
Just some ideas if the authors would be interested in exploring further down this road to get more specific answers.

We thank the reviewer for pointing this interesting and important ideas. We agree that it would be desirable to include a number of these experiments, including *At. cephalotes* in the infection experiments, but due to COVID-19 we are not able to conduct more experiments at this time, so future work will further explore this.

From the fact that the infection experiments are not included in the final two concluding paragraphs of the main text, I am assuming that the authors indeed did not find their infection experiments easy to conclusively interpret either. However, I would like to suggest to perhaps add some sort of discussion/conclusion on this aspect here.

Thanks for pointing this out, we had overlooked including it and have now added the wording "at least in part, both to (i)........ and (ii) protect them from disease organisms that might otherwise spread rapidly in their densely populated colonies." (lines 299-303, P.13).

As for my comment based on grooming behavior, this comes forth from the confusion I had, while reading the materials and methods for the infections experiment. I would suggest revising Lines 562-563 to clarify better how many ants were in each group, how many replicates there were for each group (I think 5? From 5 subcolonies?) and if these ants were kept in groups of three (meaning they could groom one another to remove spores) or by themselves (meaning it would be more difficult to groom). Further reading the methods, it also became clear that there were control groups with sham treatments. These groups should be described better (I assume a control for both with and without

biomineralization was performed for every replicate but this is not clear) and should be included in the survival curve in Figure 3.

We agree with the reviewer. As suggested, we now added more details into Method "The control for each replicate consisted of inoculating with a control solution of sterile, deionized water + 0.01% Tween 20" (lines 583-585, P.21) and "(3 individuals per sub-colony over 5 sub-colonies)" (lines 585-586, P.21) and included the control group in the survival curve in Fig.4.

As for that survival curve. It suggests that not all ants with biomineralization died within 6 days post infection. However, the main text (Line 255) mentions that all ants died within 6 days. Maybe I am misinterpreting Lines 251-255. Could the authors revise these lines to make them more clear?

We apologize for the unclear description. We meant to say that on Day 6 all ants were infected but had not yet died. We have reworded this sentence to make this clear, "On day 6 all ants had succumbed to infection and examination of workers...." (lines 262-263, P.12).

Additionally, the authors should provide more information about the fungal aspects of their infection experiment. The strain number of Metarhizium anisopliae should be mentioned. Also, the authors might want to check if this strain is still referred to as M. anisopliae since many isolates have been recognized to be specific and have, as such, have been assigned their own species names. The authors should also include culturing conditions including media used, time cultured, at what temperature, and if spores were harvested fresh before each infection experiment to assure that spores were equally viable for each replication and each group tested (did they do a spore viability test perhaps?).

We agree with the reviewer and have now added more detail to clarify the infection experiment procedure. Specifically, we added this description of the procedure into the main text: "The *Metarhizium anisopliae* var. *anisopliae* strain was isolated from dead fungus-growing ants in Gamboa, Panama, and grown on a pure medium of potato dextrose agar at room temperature until a full plate of spores was observed after around 7 days. The spores (conidia) were centrifuged, washed three times with sterile 0.05% Triton-X solution, and then harvested fresh for each infection experiment. For the infection experiments, spore suspensions were set up from recently sporulating cultures in a solution of sterile-deionized water containing 0.01% Tween 20. Spore concentration was quantified using a haemocytometer and diluted to achieve a concentration of ca. 1.00×10^7 conidiospores ml^{-1}." (lines 587-595, P.21).

Moreover, the authors mention that fungal growth was observed from cadavers of succumbed ants without biomineralization but not with. How long were these ant cadavers incubated? Under which conditions? And how many cadavers of those incubated showed growth for each group? Did they find 100% growth in cadavers without and 0% growth in cadavers with biomineralization, even though they were incubated for the same amount of time under the same conditions? This information will provide the necessary context to interpret the results that the authors report on better.

We agree with the reviewer and have added more details to the Materials and Methods section as follows: "Compared to the biomineral-present ants, fungal growth was more frequently observed

by naked eye from cadavers of succumbed ants without biomineralization at 10 days. After 2 weeks, fungal growth was observed in almost all the cadavers from both groups." (Supplementary text, P.2).

Despite these points, that I feel the authors should at least consider addressing, I really enjoyed reading this manuscript because of the incredibly exciting findings reported.

Again, thanks for the encouraging comments on our work!

Author's Response to Reviewer 3:

Li et al's article describes an investigation into the structure of the epicuticle of leaf-cutter ants. The authors show that it is covered with a thin layer of high Mg calcite, and that the formation of this phase is promoted by the organic matrix. The mineralized layer is shown to offer superior mechanical properties. I thoroughly enjoyed reading this article, where it was such a nice change to read a simple story that was so easy to follow. And I loved how the story built to an exciting climax where the importance of superior mechanical properties are tested when the ants go to war and encounter a fungus plague. I think this is an important first demonstration of biomineralization in the insect world, and I recommend publication subject to some minor changes.

We thank the reviewer for the encouraging comments on our work.

1. Sometimes the authors use terminology that I was not familiar with/ is specific to the field of "ants". As this article will also be read more generally, the authors need to explain any specific terms. Some I spotted were "obligate mutualism", "eclosion" and "callow".

We agree with the reviewer, and have added additional text to explain entomological terminology, including "eclosion (emergence of the adult from the pupal state)" and "callow adults (i.e., adults that have just emerged from the pupal stage)", lines 185, P.9, and lines 184188, P.9.

2. In the section "Morphological, structural, and chemical characteristics of epicuticular minerals" please provide a better description of the crystals (size, shape, thickness of the crystal layer etc). Currently, one has to look at the Figs to find this. It would also be useful to give information on what % of the cuticle corresponds to the mineral layer.

We agree with the reviewer and have added "The layer is composed of euhedral rhombohedral crystals with curved faces, 3-5 μm in size (Fig. 1b)" into the main text, lines 97-98, P.5.

3. In Fig 1 there is a huge image of an ant (I roughly know what these look like) but the image of the mineral film - which is the focus of the paper - is really small.

We agree with the reviewer, and thank them for pointing this out. To address this we have restructured figure 1 to be focused on the ant and biomineral layer, and adding the two

previously supplemental figures mentioned below, formerly Figs. S1 and S2, to the as the new Fig.1.

4. Fig 1b. Is the reference calcite diffractogram pure calcite? There doesn't seem to be a good fit to either calcite or dolomite. It would be more helpful if a Mg-calcite reference was used, giving a perfect match to the experimental peaks.

Several lines of evidence from the literature indicate that the 104 peak shift from calcite to dolomite is due to the Mg concentration (Graf and Goldsmith 1956 *J Geol*; Zhang et al. 2012 *Am Mineral*). That is why the Mg-calcite mineral of the ant does not exactly fit either calcite or dolomite, indicating that the biomineral is high Mg-calcite, which is further supported by the Raman and FTIR spectra Considering the current space occupied by Fig. 1, we decided not to also include a baseline reference for Mg-calcite.

5. Fig 1h is really small such that I find it hard to see the detail in the images.

We apologize for this. As mentioned in the figure legend on lines 752-753, page 25, we provide magnified images of Fig.1h in Fig. 1g in the original version. As we have restructured figs 1 & 2, and added "Fig. 2f magnifies images of Fig. 2g" into the text to clarify this (lines 139-140, p.8).

6. I found Figs S1 and S2 really useful in helping me to visualize the system. Please move to the main paper (you have space).

We that the reviewer for this great suggestion, and as previously noted, have now move Figs S1 and S2 into new Fig. 1.

7. Characterization of the mineral layer is quite difficult as it is so thin compared with the cuticle. Your data is convincing that you have a high Mg calcite. However, is it not possible that an amorphous phase is also present? This would not be surprising at such high Mg levels, and the composition of the mineral layer is likely to depend on its maturity. If so, the crystalline peaks would be weaker from a given volume of sample than if it is purely crystalline. Looking at your data it is possible that this is the case. Morphologically, the sample does not look purely crystalline, and the TEM also looks like it is a mixture.

We agree with the reviewer that, if an amorphous phase is present, the peaks would be weaker than pure crystalline peaks due to the lowering of overall crystallinity. However, because the distribution of carbonate on the ants is heterogeneous, XRD could not determine whether the changes in intensity are due to overall crystallinity or just due to the abundance of carbonate.

8. The authors often refer to "no observable Ca-Mg ordering". As far as I am aware the only Ca/Mg system where there is ordering is dolomite which is a specific phase. So, do they mean that they see no evidence of dolomite?

Yes, that's correct. We did not find any evidence of dolomite in the biomineral layer of *Ac. echinatior* ants.

9. Considering the ability of the epicuticle to promote the formation of high Mg calcite

rather than calcite, is this due to nucleation on the specific surface, or soluble proteins released into the solution?

We thank the reviewer for pointing out our lack of clarity about this. As mentioned in lines 376-377 in the original paper, “Filtered solution and ants were air dry for XRD and SEM characterization”, but in fact what we meant to write was “Filters and ants were air dried . . .” We have corrected this sentence to read “Filters and ants were air dried for XRD and SEM characterization” (lines 391-392, P.17). Given the fact that high Mg-calcite mineral is only be found on the surface of the ant integument, we think it is unlikely that soluble proteins are released into the solution.

Reviewer #1 (Remarks to the Author):

I thank Li & colleagues for taking the time to formulate point specifics answer addressing my previous remarks. However, I fear that important advises have been ignored and I recommend strong actions from the authors. The manuscript in its current form still suffers from a large bias in terms of conclusion drawing, and thus does not meet the quality criteria for publishing in Nat. Com. A point to point explaination will be developped hereafter.

Major Remark I

^{(A) We are grateful for the reviewer's interest in ant cuticular hardness and we acknowledge that there is a great amount of interesting additional data for us to collect and explore but, again, gathering and adding more information to the present paper is not possible due to COVID19. We also respectfully point out that the reviewer must have missed that we present Ac. echinatior workers without the biomineral layer as our main control group (Fig. 4a). […] (B)We agree with the reviewer that it would be desirable to include Atta workers in entomopathogenic fungal infection experiments, but due to COVID-19 we are not able to conduct more experiments at this time, so future work will follow up on this. We also point out that our goal was not to determine whether the biomineral armor provides a competitive advantage to a species with biomineral armor vs. a second species without armor, but rather whether it confers protection from entomopathogenic fungi in Acromyrmex echiniator}.

I want to make a rational case here on why the design of these experiments is currently inadequate to fully support authors' conclusions. Considering that ants all belong to a single family, data on other insects are interesting but also evolutionary distant. Under that light, author’s true meaningful comparison for cuticle hardness experiments becomes a two-species one inside the Myrmecine subfamily (Atta vs Acromyrmex). For Immunity, it becomes biomineralized workers vs non-biomineralized. Please refer to the following on why this approach is inherently biased and should be avoided at all cost:

“Garland Jr, T., & Adolph, S. C. (1994). Why not to do two-species comparative studies: limitations on inferring adaptation. Physiological Zoology, 67(4), 797-828.”

Consider this, for example.

(1) All Myrmecine ants may have evolved very hard cuticle as compared to other insects. Authors cannot know since they did not test for any species outside of the Attine tribe.

(2) It may be that softer cuticle secondarily evolved alongside the whole Attine tribe, and that biomineralization is only a case-specific way to make up for what has been lost. In which case, you would observe Acromyrmex biomineralized cuticle being no more hard than the one of other Myrmecine ants (with the exception of A. cephalotes who stayed squishy). This would drastically change the view you need to adopt on this finding. Authors cannot know for sure since they lack the appropriate controls, both in cuticle hardness and fighting experiments.

(3) Maybe biomineralized A. echinatior workers remains more sensitive to fungal infection than other species, making such an armor rather useless for overall immunity purposes. It is impossible to tell without controls.

Thus, you cannot say based on a two species comparison that biomineralization makes A. echinatior an overall better fighter, or more immune to pathogens. You can only say that you highlight a mechanism that strengthens its cuticle and seems to reduce infection likeliness, lacking much needed contextual data to safely infer any real adaptative values. Hence a large gap exists between what is insinuated thorough the discussion (better at fighting and immunity), and the reality of your data. Furthermore, as far as the biological function of such an armor may go, you only considered a couple of all its potential drivers, and when you did, you frame experiments in such a way that they do not allow you to extract meaningful adaptative value safely. This is why having third and fourth species as control matters when inferring the use of adaptative traits (See Garland et al., 1994).

The current manuscript can thus only be taken as an interesting description of a novel mechanism to strengthen ant cuticle. Further biological inference on its functions under this current experimental framework would be unwise. If authors cannot consider performing additional experiments with the proper controls, they should at the very least reframe their interpretations of functional morphology to reflect their uncertainty about adaptative values (line 242-246; 290-299). They should also clearly mention that they cannot know if such an armor truly makes Acromyrmex a better fighter in the ant kingdom, or even better at coping with fungal infection altogether as compared to other ants, which is unfortunately still insinuated when reading the discussion for the lack of being clearly stated.

(II) Minor remarks

<sup>(a) V. Minor remarks Introduction Line 71: A mature leaf-cutter ant colony comprises a "superorganism" with 100,000 to > 5 million workers, a single queen. Acromyrmex colonies have been shown to both be smaller (Wetterer, 1995) and, for some species, including A. echinatior which is the focus of the present study, facultatively polygynous (Bekkevold et al., 1995). Please nuance.

As suggested, we add “including those of Acromyrmex,” (lines 86-87, P.5) into introduction.</sup>

This answer is likely a mistake, since it is a replicates of the one formulated for the following minor remark and do not address the present point. Please give the correct range of A. echinatior colony size, and explicitly state that the species is facultatively polygynous.

<sup>(b) Line 86: Many species of fungus-growing ants are variably covered with a whitish granular coating, uniformly distributed on their otherwise dark-brown cuticles. Please give a few examples.

As suggested, we add “, including Acromyrmex echinatior” (lines 91-92, P.5) into introduction.</sup>

Since authors state that many species are likewise covered, it spikes the curiosity of readers. Accordingly, please explicitly give a few examples of relative fungus-farming ants presenting a similar whitish coating.

<sup>(c)Lines 212-215: Please frame results of cuticle thickness in their broader evolutionary perspective in ants using data from Peeters et al., 2017. It might be worth mentioning that some subfamilies (Ponerine) do have even thicker exoskeletons than Acromyrmex, and likely harder too as a result, but no evidence points out toward biomineralization in those species so far.

As noted above, we are grateful for the reviewer's interest in ant cuticular hardness and thickness; future work will explore this. </sup>

Including cuticle thickness for A. cephalotes in the present manuscript is needed for a relevant comparison of cuticle hardness between the two species. Please extract value from your own dataset or from Peeters et al., 2017 and compare it with A. echinatior cuticle’s thickness in the manuscript.

Reviewer #2 (Remarks to the Author):

Thank you for addressing all the points raised. I have no further comments or concerns.

Reviewer #3 (Remarks to the Author):

The authors have answered some of my questions, but others not.

(1) I really would like you to match the XRD diffractogram in Fig 2 with magnesian calcite of the appropriate composition. You can readily model what the pattern would be. Currently, the data are matched to two phases that don’t exist in the sample - dolomite and pure calcite. If you don’t have space remove the patterns of the phases that aren’t in the sample, and show the correct pattern instead.

(2) “However, because the distribution of carbonate on the ants is heterogeneous, XRD could not determine whether the changes in intensity are due to overall crystallinity or just due to the abundance of carbonate.”

I don’t understand what is meant here. Both crystalline $CaCO_3$ and ACC have the same Ca: CO_3 ratio. Surely you should at least mention in the text that it is possible that there is some ACC present?

A good way of proving whether a sample is partially crystalline is to try to induce crystallisation. You could try heating/ irradiation with the electron beam in a TEM.

(3) “The authors often refer to “no observable Ca-Mg ordering”.

As far as I can see you haven’t changed the text in any way to clarify to the reader that you mean no dolomite is present. I would imagine that many readers will not be aware that dolomite is the only Ca/$MgCO_3$ phase that is ordered.

(4) I asked: “Considering the ability of the epicuticle to promote the formation of high Mg calcite rather than calcite, is this due to nucleation on the specific surface, or soluble proteins released into the solution?”

I find the answer unsatisfactory. Yes, the substrate clearly offers an attractive surface for nucleation. However, the substrate will only affect nucleation (eg number and orientation of the crystals) and not their final morphologies. That is why I asked whether there are additional organics in the solution (released from the substrate) that are modifying the shape. These crystals do not have morphologies I would expect on growth from an organic-free solution.

AUTHOR'S RESPONSE TO REVIEWER 1:

Major Remark I

(A) We are grateful for the reviewer's interest in ant cuticular hardness and we acknowledge that there is a great amount of interesting additional data for us to collect and explore but, again, gathering and adding more information to the present paper is not possible due to COVID19. We also respectfully point out that the reviewer must have missed that we present Ac. echinatior workers without the biomineral layer as our main control group (Fig. 4a). [...] (B)We agree with the reviewer that it would be desirable to include Atta workers in entomopathogenic fungal infection experiments, but due to COVID-19 we are not able to conduct more experiments at this time, so future work will follow up on this. We also point out that our goal was not to determine whether the biomineral armor provides a competitive advantage to a species with biomineral armor vs. a second species without armor, but rather whether it confers protection from entomopathogenic fungi in Acromyrmex echiniator.

I want to make a rational case here on why the design of these experiments is currently inadequate to fully support authors' conclusions. Considering that ants all belong to a single family, data on other insects are interesting but also evolutionary distant. Under that light, author's true meaningful comparison for cuticle hardness experiments becomes a two-species one inside the Myrmecine subfamily (Atta vs Acromyrmex).

We respectfully disagree. Our hypothesis, relating to this part of our manuscript, is simply that the biomineral layer provides a substantial increase in cuticle hardness for *Ac. echinatior.* The only true control for this is *Ac. echinatior* without the biomineral layer. We are perplexed by the reviewer arguing that the 'true meaningful comparing for cuticle hardness' is comparing cuticle hardness to other species of ants. Simply put, would the reviewer expect us to conclude that *the biomineral increases cuticle hardness* if we compared *Ac. echinatior* ants with biomineral to other species of ants, ants that may or may not have a biomineral layer? In such a comparison, it would be impossible to determine if increases in cuticle hardness were conferred by the biomineral layer or just differences in the exoskeleton. We point out that this is the first discovery of a biomineral layer in any insect.

For Immunity, it becomes biomineralized workers vs non-biomineralized. Please refer to the following on why this approach is inherently biased and should be avoided at all cost:

"Garland Jr, T., & Adolph, S. C. (1994). Why not to do two-species comparative studies: limitations on inferring adaptation. Physiological Zoology, 67(4), 797-828."

We agree that comparative studies focusing on two species are problematic. However, our paper is not a comparative study; rather it is a study of *Ac. echinatior* ants with and without biominerals, involving properly controlled experimental manipulations to support our findings.

We believe that the addition of other insects is not central to our findings nor required for our conclusions.

Consider this, for example.
(1) All Myrmecine ants may have evolved very hard cuticle as compared to other insects. Authors cannot know since they did not test for any species outside of the Attine tribe.

According to Peeters et al. (2017), which the reviewer cites, even within the ants, the thickest cuticles have evolved in the poneroid subfamilies and genera rather than in the Myrmicinae (e.g., Peeters et al. 2017, Figure 3). Also according to Peeters et al. (2017), cuticle thickness in ants is much more correlated with body size than it is with phylogeny. And, as Peeters et al. (2017) point out, thicker cuticles come at the expense of evolutionary constraints and energetic tradeoffs: ". . . cost savings from a thinner cuticle may be miniscule for one individual, but savings are considerably amplified in the populous colonies of social insects." Thus, based on the data in Peeters et al. (2017), we feel there is no justification to speculate that all myrmicine ants ‘have evolved very hard cuticles compared to other insects’. In fact, it is widely recognized that most beetles have harder cuticles than other insects, and they are still not ‘very hard’ (see Figure 4A), at least as compared to *Ac. echinatior* workers with the integumental biomineral layer.

(2) It may be that softer cuticle secondarily evolved alongside the whole Attine tribe, and that biomineralization is only a case-specific way to make up for what has been lost. In which case, you would observe Acromyrmex biomineralized cuticle being no more hard than the one of other Myrmecine ants (with the exception of A. cephalotes who stayed squishy). This would drastically change the view you need to adopt on this finding. Authors cannot know for sure since they lack the appropriate controls, both in cuticle hardness and fighting experiments.

The reviewer appears to be suggesting, without any empirical support, that compared to other insects, myrmicine ants evolved very hard cuticles, then *Acromyrmex* and *Atta* ants secondarily evolved softer cuticles. Then, *Acromyrmex echinatior* evolved the incredibly unusual ability to form a biomineral layer composed of high-magnesium calcite to compensate for the loss of the ancestral very hard cuticle. Although it is possible to postulate any number of such non-parsimonious scenarios, they do not have any bearing on our conclusions, as our study is simply focused on showing that *Ac. echinatior* ants with the biomineral armor are substantially harder than *Ac. echinatior* ants without the biomineral armor. Simply put, reconstructing evolutionary patterns of cuticle hardness and the biomineral layer across ants and insects is not part of our study. In fact, our study is the first report of biomineral armor in any insect. Whether biomineral armor is common in insects or not will be determined by future research.

(3) Maybe biomineralized A. echinatior workers remains more sensitive to fungal infection than other species, making such an armor rather useless for overall immunity purposes. It is impossible to tell without controls.

Again, we point out that the proper experimental and control groups to test our hypothesis are ants with and without biomineral armor, respectively.

Thus, you cannot say based on a two species comparison that biomineralization makes A. echinatior an overall better fighter, or more immune to pathogens. You can only say that you highlight a mechanism that strengthens its cuticle and seems to reduce infection likeliness, lacking much needed contextual data to safely infer any real adaptative values. Hence a large gap exists between what is insinuated thorough the discussion (better at fighting and immunity), and the reality of your data. Furthermore, as far as the biological function of such an armor may go, you only considered a couple of all its potential drivers, and when you did, you frame experiments in such a way that they do not allow you to extract meaningful adaptative value safely. This is why having third and fourth species as control matters when inferring the use of adaptative traits (See Garland et al., 1994).

As noted above, our paper is not a comparative study nor an attempt to reconstruct evolutionary patterns of cuticle hardness and biomineralized armor in ants and insects.

The current manuscript can thus only be taken as an interesting description of a novel mechanism to strengthen ant cuticle.

We respectfully point out that this statement constitutes an acknowledgement by the reviewer, contrary to their statements above, that we have shown that the biomineral layer strengthens the ant cuticle. Reporting the first discovery of biomineral armor in any insect and demonstrating that it strengthens the ant cuticle are the two primary findings of our study.

Further biological inference on its functions under this current experimental framework would be unwise. If authors cannot consider performing additional experiments with the proper controls, they should at the very least reframe their interpretations of functional morphology to reflect their uncertainty about adaptative values (line 242-246; 290-299). They should also clearly mention that they cannot know if such an armor truly makes Acromyrmex a better fighter in the ant kingdom, or even better at coping with fungal infection altogether as compared to other ants, which is unfortunately still insinuated when reading the discussion for the lack of being clearly stated.

Yes, we agree that we cannot compare the integumental hardness of *Acromyrmex echinatior* to that of other ants that we did not measure. We can, however, compare the integumental hardness of *Acromyrmex echinatior* to that of *Atta cephalotes*, which we did measure. We can also compare the integumental hardness of *Acromyrmex echinatior* workers with and without the biomineral, which we also measured. We also agree with the reviewer that we cannot prove that the functions we explored are the functions that drove the evolution of the biomineral layer. Instead, we can only show that they are plausible functions. That is why we are careful to say that we have demonstrated -- but not proven -- possible functions of the biomineral layer. We have done this by comparing the performance of *Ac. echinatior* vs. *Atta cephalotes* in aggressive encounters and by comparing the resistance to fungal pathogens of the cuticles of workers with and without the biomineral layer. As we have already said, we are unfortunately unable to conduct any further experiments and/or comparisons in the lab at this time due to the covid-19 pandemic. Nevertheless, to make it unambiguous, we have now added ". . . even though more ant species need to be further investigated." (lines 262-263, page 12), and the sentence "Further, given that fungus-growing ants are among the most extensively studied tropical insects, our finding raises the intriguing possibility that high-magnesium calcite biomineralization may be

more widespread in insects than previously suspected, suggesting a promising avenue for future research." (line 292-296, page 13).

(II) Minor remarks

(a) V. Minor remarks Introduction Line 71: A mature leaf-cutter ant colony comprises a "superorganism" with 100,000 to > 5 million workers, a single queen. Acromyrmex colonies have been shown to both be smaller (Wetterer, 1995) and, for some species, including A. echinatior which is the focus of the present study, facultatively polygynous (Bekkevold et al., 1995). Please nuance. As suggested, we add "including those of Acromyrmex," (lines 86-87, P.5) into introduction. This answer is likely a mistake, since it is a replicates of the one formulated for the following minor remark and do not address the present point. Please give the correct range of A. echinatior colony size, and explicitly state that the species is facultatively polygynous.

We have altered this sentence to read: "A mature *Atta* leaf-cutter ant colony comprises a "superorganism" with > 5 million workers and a single queen. *Acromyrmex* leaf-cutter colonies vary in size, generally within the range of 15,000 to 100,000 workers (Wetterer, 1995; Weber, 1972), and in some species colonies may have more than one queen. *Acromyrmex echiniator*, the focus of the present study, has a mean colony size of 137,500 workers and is facultatively polygynous (Bekkevold et al., 1995; Ferguson-Gow et al., 2014).", lines 74-80, page 4.

(b) Line 86: Many species of fungus-growing ants are variably covered with a whitish granular coating, uniformly distributed on their otherwise dark-brown cuticles. Please give a few examples. As suggested, we add ", including Acromyrmex echinatior" (lines 91-92, P.5) into introduction. Since authors state that many species are likewise covered, it spikes the curiosity of readers. Accordingly, please explicitly give a few examples of relative fungus-farming ants presenting a similar whitish coating.

We have added the following to the text: "Many species of fungus-growing ants are variably covered with a whitish granular coating, uniformly distributed on their otherwise dark-brown cuticles, including, in addition to *Acromyrmex echinatior*, some species of *Trachymyrmex* and *Sericomyrmex*.", lines 94-97, page 5.

(c)Lines 212-215: Please frame results of cuticle thickness in their broader evolutionary perspective in ants using data from Peeters et al., 2017. It might be worth mentioning that some subfamilies (Ponerine) do have even thicker exoskeletons than Acromyrmex, and likely harder too as a result, but no evidence points out toward biomineralization in those species so far. As noted above, we are grateful for the reviewer's interest in ant cuticular hardness and thickness; future work will explore this. Including cuticle thickness for A. cephalotes in the present manuscript is needed for a relevant comparison of cuticle hardness between the two species. Please extract value from your own dataset or from Peeters et al., 2017 and compare it with A. echinatior cuticle's thickness in the manuscript.

We have added the following text: "These cuticle thickness values agree with those reported by Peeters et al. 2017.", lines 233-234, page 11.

AUTHOR’S RESPONSE TO REVIEWER 2

Thank you for addressing all the points raised. I have no further comments or concerns.

Thanks for the encouraging comments on our work!

AUTHOR’S RESPONSE TO REVIEWER 3

The authors have answered some of my questions, but others not.

(1) I really would like you to match the XRD diffractogram in Fig 2 with magnesian calcite of the appropriate composition. You can readily model what the pattern would be. Currently, the data are matched to two phases that don’t exist in the sample - dolomite and pure calcite. If you don’t have space remove the patterns of the phases that aren’t in the sample, and show the correct pattern instead.

We thank the reviewer for this useful suggestion. As suggested, we now add the Mg-calcite reference in Fig 2.

(2) “However, because the distribution of carbonate on the ants is heterogeneous, XRD could not determine whether the changes in intensity are due to overall crystallinity or just due to the abundance of carbonate.”

I don’t understand what is meant here. Both crystalline $CaCO_3$ and ACC have the same Ca: CO_3 ratio. Surely you should at least mention in the text that it is possible that there is some ACC present?

A good way of proving whether a sample is partially crystalline is to try to induce crystallisation. You could try heating/ irradiation with the electron beam in a TEM.

Sorry that this was unclear. As suggested, we have now added this additional information about heat-treating and irradiating biomineral-bearing ants to the text "To investigate the presence of amorphous calcium carbonate within the biomineral cuticular layer, we heat-treated or irradiated biomineral-bearing ants, and acquired XRD and TEM-SAED data before and after either treatment. We did not find evidence of increased crystallinity in either the heat or radiation treatments in either x-ray or electron diffraction.", lines 133-137, page 7.

(3) “The authors often refer to “no observable Ca-Mg ordering”.

As far as I can see you haven’t changed the text in any way to clarify to the reader that you mean no dolomite is present. I would imagine that many readers will not be aware that dolomite is the only Ca/$MgCO_3$ phase that is ordered.

Thanks for pointing this out. In the original paper, we chose to take what we regard as a conservative approach, using the term ‘no observable Ca-Mg ordering’ to refer to the biomineral cuticular layer on the ants. The reason for that is to avoid confusion of the high-Mg-calcite with dolomite and/or partially ordered dolomite, which as the reviewer has mention is the only phase in Ca-Mg carbonate system that has cation ordering. We have now added the text " (i.e., no evidence for dolomite, the only Ca-Mg carbonate phase with cation ordering)" (lines 129-130,

page 7), to make this more clear.

(4) I asked: "Considering the ability of the epicuticle to promote the formation of high Mg calcite rather than calcite, is this due to nucleation on the specific surface, or soluble proteins released into the solution?"

I find the answer unsatisfactory. Yes, the substrate clearly offers an attractive surface for nucleation. However, the substrate will only affect nucleation (eg number and orientation of the crystals) and not their final morphologies. That is why I asked whether there are additional organics in the solution (released from the substrate) that are modifying the shape. These crystals do not have morphologies I would expect on growth from an organic-free solution.

Sorry that this was unclear. We agree with the reviewer that, based on our *in vitro* synthesis, the ability of the epicuticle to promote the formation of high-Mg-calcite is most likely due to nucleation on the specific surface, which promotes the incorporation of Mg^{2+} into calcite structure, leading to the formation of Mg-calcite. We also agree with the reviewer that the crystal morphology is consistent with a solution containing organic molecules. Our solution did not contain any organic solutes, other than molecules that originate from the ant thorax exoskeletons, which provided the substrate for the synthesis experiment. We have added this information to the text, ", in particular the synthetic crystal morphology is consistent with a solution containing organic molecules that likely originated in the ant thorax exoskeletons," (lines 185-187, page 9).

REVIEWERS' COMMENTS:

Reviewer #3 (Remarks to the Author):

I am now happy that the authors have addressed all of my queries and recommend publication in Nature Comms.
